# *Bacillus pumilus* and *Bacillus subtilis* Promote Early Maturation of Cecal Microbiota in Broiler Chickens

**DOI:** 10.3390/microorganisms9091899

**Published:** 2021-09-07

**Authors:** Muhammad Bilal, Caroline Achard, Florence Barbe, Eric Chevaux, Jennifer Ronholm, Xin Zhao

**Affiliations:** 1 Department of Animal Science, McGill University, Sainte-Anne-de-Bellevue, QC H9X 3V9, Canada; bilal.orakzai@hotmail.com (M.B.); jennifer.ronholm@mcgill.ca (J.R.); 2 Lallemand Animal Nutrition, 31702 Blagnac, France; cachard@lallemand.com (C.A.); fbarbe@lallemand.com (F.B.); echevaux@lallemand.com (E.C.); 3 Department of Food Science, McGill University, Sainte-Anne-de-Bellevue, QC H9X 3V9, Canada

**Keywords:** probiotics, microbiome, maturity, *Bacillus subtilis*, *Bacillus pumilus*, gut microbiota, Ruminococcaceae, Lachnospiraceae

## Abstract

Mature and stable intestinal microbiota in chickens is essential for health and production. Slow development of microbiota in young chickens prolongs the precarious period before reaching mature configuration. Whether probiotics can play a role in the early maturation of intestinal microbiota is unknown. To address this, day-old chicks were assigned into six groups: NC (basal diet), PC (virginiamycin), low (BPL) and high-dose (BPH) of *Bacillus pumilus,* and low (BSL) and high-dose (BSH) of *Bacillus subtilis*. Cecal contents at days 7, 14, 28 and 42 were used to analyze the treatment and time effects on the diversity and composition of microbiota. Overall, the alpha diversity was significantly decreased in the NC group between days 7 and 14, while this decline was prevented in the *Bacillus subtilis* probiotic (BSL and BSH) and even reversed in the BPH group. The beta-diversity showed significant responses of microbial communities to probiotics in first two weeks of life. Analyses of the abundance of microbiota reflected that members of the family Ruminococcaceae (*Ruminnococcus*, *Oscillospira*, *Faecalibacterium*, *Butyricicoccus,* and *Subdoligranulum*), which were dominant in mature microbiota, were significantly higher in abundance at day 14 in the probiotic groups. Conversely, the abundance of genera within the family Lachnospiraceae (*Ruminococcus*, *Blautia,* and *Coprococcus*) was dominant in early dynamic microbiota but was significantly lower in the probiotic groups at day 14. The *Lactobacillus* and *Bifidobacterium* abundance was higher, while the Enterobacteriaceae abundance was lower in the probiotic groups. In summary, the probiotics efficiently helped the cecal microbiota reach mature configuration earlier in life. These results could be used for the future manipulation of microbiota from the perspective of improving poultry performance.

## 1. Introduction

Poultry is a growing contributor to human dietary protein intake and is an important contributor to feeding a growing human population. Poultry production increased from 9 to 132 million tons between 1961 and 2019 [1]. The poultry sector is estimated to grow at an annual rate of 2–3% between 2015 and 2030, the highest growth rate in the livestock sector [2]. The tremendous advance in the poultry production system during the last 50 years has been achieved through improvements in genetics, management, and nutrition. Among the improvement of nutrition, the use of feed additives has increased and has contributed to the success in current broiler production. Probiotics are among the most researched feed additives and show promising results for production and health parameters [3]. Probiotics produce their effects through different mechanisms. Our laboratory has previously demonstrated the role of probiotics in the alleviation of pathogen-associated inflammation [4] and disrupted intestinal permeability [5].

The intestinal microbiota plays a key role in immune development, and its homeostatic interactions with the host are now well established [6]. Intestinal microbiota can be influenced by both environmental- and host-related factors. Host-related factors such as age, sex, breed [7], and host immune system [8] influence the structure and composition of microbiota. For example, the immune system has the ability to change the configuration of the microbiota by determining which bacteria are allowed to colonize the gut and which will be excluded via secreted antibodies [9]. The age of the host also affects the diversity and stability of the microbiota. In broiler chickens, the intestinal microbiota is dynamic during the first few weeks of life, which is followed by a mature and stable microbiota [10]. Hartog et al. [11] observed a marked decrease in the microbial diversity in the early weeks of layer chickens followed by a stable microbiota after 42 days of life. They associated this early life decrease followed by stability in microbial diversity with host immune response, which gradually matures and stabilizes with passage of time. We recently observed that immune and gut health responses to the probiotic groups in broilers were different at different stages of life, which were shown to be significant at day 14 and insignificant at day 42 [12]. Intestinal perturbation in microbiota can be induced by exogenous factors such as antibiotics. Probiotics have been used to prevent antibiotic-induced dysbiosis [13]. However, whether age-related low microbial diversity in early life can be improved with probiotics is less studied in broiler chickens.

The maturation of microbiota is important for optimal host metabolism [14] and immune development [15]. A mature microbiota has higher resilience to different stress factors [16]. The microbial population of phylum Firmicutes is the main and dominant group in chicken intestine. Lachnospiraceae and Ruminococcaceae are two main families in the phylum Firmicutes that can be found in chickens [10]. Members of Lachnospiraceae are considered biomarkers of an early and immature microbiota [10,17], while members of the family Ruminococcaceae are in higher abundance in mature stable microbiota [18,19]. It is worth noting that for more than half of the production period, the microbiota of broiler chickens is developing and is vulnerable to external stressors. Dietary interventions in the microbiota are likely to be more successful if they can promote the early maturation of the microbiota, particularly with respect to members of Lachnospiraceae and Ruminococcaceae. Recently, contact with adult hens [20] and inoculation with adult-derived microbiota [21] showed an acceleration in the maturation of intestinal microbiota. Though several studies have evaluated the effect of probiotics and antibiotics on microbiota [6], investigations of whether these interventions influence the maturity of microbiota is still relatively scarce.

The main objective of this study was to evaluate the impact of *Bacillus* probiotics, *B. pumilus,* and *B. subtilis* on microbial diversity and maturity in terms of changes in the composition of the families Lachnospiraceae and Ruminococcaceae in cecal microbiota at different stages of life in broiler chickens. *Bacillus*-based probiotics were used in this study since they have an advantage over other probiotics due to their ability to form spores, which increases their survivability in feed processing and in the gastrointestinal tract.

## 2. Materials and Methods

### 2.1. Birds, Diet and Experimental Design

A total of 2073 one-day old male Cobb 500 chicks were obtained from a local hatchery (Grains Natures, Roxton Falls, QC, Canada) and were randomly divided into 36 pens (6 pens/treatment). These broilers were assigned to 6 treatments and were grown for 42 days. The dietary treatments included a standard basal diet as a negative control (NC), a basal diet with antibiotic growth promoter as a positive control (PC) (Virginiamycin at 16.5 mg/kg of feed), a basal diet with a low-dose of *B. pumilus* (3 × 10^8^ CFU/kg of feed) (BPL), a basal diet with a high-dose of *B. pumilus* (1 × 10^9^ CFU/kg of feed) (BPH), a basal diet with a low-dose of *B. subtilis* (3 × 10^8^ CFU/kg of feed) (BSL), and a basal diet with a high-dose of *B. subtilis* (1 × 10^9^ CFU/kg of feed) (BSH). The management of broilers including the composition of the basal diet with monensin as the anticoccidial agent was described previously [12]. The probiotics were provided by Lallemand SAS, France. The study protocol was approved by the Animal Care Committee of McGill University.

### 2.2. Sample Collection and DNA Extraction

The baseline data at day one was not collected since the cecal content is minimal at this age. Instead, the first sampling was conducted on day 7. In addition, we took measures to avoid initial biases for all of the groups, such as all of the chicks being form the same source of hatchery, all of the chicks being of the same sex (male broilers) of equal average weight, the randomized allocation of the chicks to the pens, and the randomized allocation of the pens to the treatments. At days 7, 14, 28, and 42, one bird per pen (*n* = 6/group) was randomly selected and was euthanized by cervical dislocation, and both ceca were removed to obtain the cecal contents. The cecal contents were collected in cryovials, snap-frozen in liquid nitrogen, and stored at −80 °C. DNA from these samples were isolated through a DNeasy PowerSoil Pro Kit (QIAGEN, Montreal, Canada) with a bead-beating mechanical lysis step to increase the DNA yield. After quality checks through a spectrophotometer, DNA was stored at −80 °C for further use.

### 2.3. Sequencing and Data Analysis of Cecal Microbial Community

Illumina MiSeq (Illumina, San Diego, CA, USA) paired-end sequencing was performed to determine the bacterial community composition of each sample using the 548F and 806R primers for the V4 region of the 16S rRNA gene amplicon library preparation. The MiSeq sequencing was performed according to standard Illumina protocol using a dual-indexing strategy for multiplexed sequencing [22]. Raw sequencing data were received as the 250-bp length of each pair of the reads in FASTQ format for further processing. The Illumina data were analyzed with QIIME2 software version 2019.10.0 [23]. The reads were checked for quality and were subjected to a denoising method for the removal of low-quality reads and chimeras and for the correction of sequencing errors through the DADA2 plugin with default parameters. The phylogenetic tree was constructed through the q2-phylogeny plugin and the taxonomy was assigned using the q2-feature-classifier plugin [24] through a pre-trained Naïve Bayes classifier based on the Greengenes v. 13_8 database. Alpha and beta diversity analyses were performed at a sequencing depth of 12,850 using the QIIME2 alpha and beta diversity plugins. Different metrics, such as observed operational taxonomic units (OTU), Pielou, and Shannon, were used to assess alpha diversity. The comparison of the alpha diversity metrics among the treatments was made through the two-way ANOVA test with the groups, time, and their interaction, which was followed by the Sidak test as a post hoc test for multiple comparisons. Beta diversity, a metric used for the comparison of microbial diversity between samples, was calculated with a weighted UniFrac metric. The results were tested at each time point with a PERMANOVA test, and the multiple-test correction was completed with the Benjamini–Hochberg FDR method (q-values < 0.05) in order to assess the community differences between the groups. Taxa plots were generated using the q2-taxa plugin (https://github.com/qiime2/q2-taxa, accessed 15 May 2021) to visualize the differences in the treatment groups at the phylum level. The relative abundance of microbiota between NC and other the treatments at the family and genus levels at days 7, 14, 28, and 42 was generated and ranked through Songbird software [25]. These ranked differentials were used to pick suitable reference frames based on their presence across the most samples as the denominator for the log-ratio test using Qurro software, version v0.5.0, Knight lab, University of California, San Diego, CA USA [26]. The log ratios were calculated between the observed features and the taxon used as a reference to avoid bias associated with the analysis of compositional or relative abundance data. This method provides the opportunity to reveal microbial changes without the need to estimate the total microbial load [27]. In this study, the Ruminococcaceae was used as a reference frame for the features of all of the other microbial families, while Lachnospiraceae was used as a reference frame for the features of family Ruminococcaceae, unless different reference frames were indicated. Qurro-generated log ratios at days 7, 14, 28, and 42 were further analyzed through one-way ANOVA followed by Duncan’s test as a post hoc test for multiple comparisons.

## 3. Results

### 3.1. Sequencing Data

From the 144 samples, a total of 15,772,526 sequences were obtained, with mean of 109,531 sequences per sample. After DADA2 quality control processes, 9,317,770 sequences with a mean of 64,707 sequences per sample were retained. The samples were rarefied at 12,850 sequences per sample for even depth of analysis. The low read samples were removed, and the remaining 108 samples that reached the saturation plateau of the rarefaction curve were included for further analyses.

### 3.2. Probiotics Improve the Cecal Microbial Alpha Diversity in Young Chickens

In order to investigate the effects of supplementing probiotics on gut microbiota, the microbial richness, evenness, and diversity were examined for the cecal microbiota on days 7, 14, 28, and 42. The interaction terms (Groups * Time) for richness, evenness, and diversity were significant (*p* < 0.05) and were included in the analyses. The microbial richness was not different among treatments at days 7, 28, and 42, but significant improvement (*p* < 0.05) was observed in all of the probiotic groups at day 14 when compared to the PC and NC groups (Table 1a). Looking at the different time points (Table 1a), microbial richness significantly increased earlier in the BPL and BSH groups between days 7 and 14 and reached their peaks at day 28, while microbial richness in the BSL and BPH groups significantly increased between days 14 and 42 and reached their highest values at days 28 (BPH) and 42 (BSL). A significant increase in the species richness of the NC and PC groups also happened between days 14 and day 42.

The effects of the treatments on the evenness of the microbiota were evaluated through the Pielou index (Table 1b). The evenness of the microbiota in all of the probiotic groups at day 7 was comparable to the NC group but was significantly greater than the PC group, except for the BPH group. A significant difference was also observed between the BPH and BSH groups and between the NC and PC groups at day 7. At day 14, the evenness of the microbiota in all of the probiotic groups were comparable to the NC group, except for the BPL group, where the microbiota was significantly lower than it was in the NC and BPH groups. The evenness at days 28 and 42 was comparable among all of the groups except for that the evenness in the BSH group was significantly lower than it was in the PC group at day 28. While comparing the treatment groups at different time points (Table 1b), the microbial evenness significantly decreased in the NC and BPL groups between days 7 and 14 (*p* < 0.05), but later it was improved in the BPL group by day 28. The evenness in the BPH and BSL groups remained the same between days 7 and 14 and later significantly decreased between days 14 and 28 (BPH) and days 28 and 42 (BSL). The evenness of the BSH and PC groups were similar between days 7 and 14 but significantly increased in the PC group on day 28, while the evenness was decreased in the BSH group on days 28 and 42.

The Shannon index, which combines the effects of richness and evenness, was used to assess the changes in the alpha diversity of the microbiota in the different groups (Table 1c). The diversity of all of the probiotic groups at day 7 was equivalent to that of the NC group but was significantly greater (BPL, BSL and BSH) than that of the PC group. At day 14, the diversity in the probiotic groups was significantly higher than the NC (BPH) and PC (BPH and BSH) groups. At day 28, the microbial diversity in all of the probiotic groups became equivalent to the NC and PC groups, and this trend continued until day 42. While comparing the microbial diversity of the treatment groups among different time points (Table 1c), a significant decline was seen in the NC and BPL groups (*p* ≤ 0.05) between days 7 and 14, which significantly increased by day 28 and remained the same during rest of the study period. However, this decline was prevented in the *B. subtilis* probiotic groups (BSL and BSH) between days 7 and 14 and remained unaffected through rest of the study period. The BPH group showed a significant increase in diversity between day 7 and 14 and remained stable until day 42. The PC group remained unchanged until day 14, showed a significant increase in diversity between days 14 and 28, but the diversity significantly declined in the period between days 28 and 42 (*p* < 0.05).

### 3.3. Probiotics Affect the Cecal Microbial Beta Diversity in Young Chickens

To study the changes in microbial responses to probiotics at different stages of life, a weighted UniFrac metric was used to analyze the beta diversity of the microbial communities across treatments and at different time points. The beta diversity of the BPH and BSH groups at day 7 was significantly different from other groups (Table 2a), while at day 14, all of the treatment groups were significantly different from each other, except for the BPH and BSH groups, which were not statistically different from each other (Table 2b). At day 28, differences among the microbiota from the different treatment groups became less prominent, except between the NC group and the BPL, BSL, and BSH groups. The BSL and BSH groups were also different from the BPL group (Table 2c). The samples from the different treatment groups overlapped at day 42 (Table 2d), and significant differences were only seen between the PC group and the NC and BPL groups. While comparing the microbial beta diversity of the treatment groups among time points (Table 2e), it was observed that the microbiota in the probiotic groups BPL, BPH, and BSH was significantly different at day 14 than it was at day 7 and at day 28 than it was at day 14, but no significant difference seen between microbiota at days 28 and 42. The NC and BSL groups were different at all time points (days 14, 28, and 42) when compared to the previous time points. The PC group showed significant differences in the microbiota between days 7 and 14 followed by insignificant changes in the microbiota between days 14 and 28, with a significantly different set of microbiota being presented again at day 42. These results again showed that changes in the cecal microbiota for the probiotic groups were visible in the early weeks of life, which were the most prominent at day 14, and these became insignificant after day 28.

### 3.4. Probiotics Change Composition of Cecal Microbiota in Broiler Chickens

**Figure 1:**
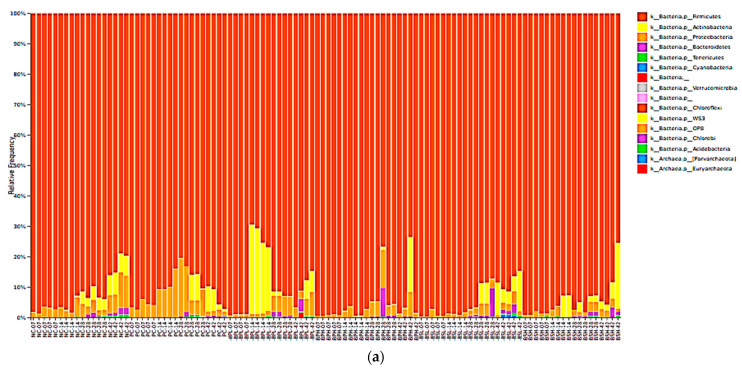

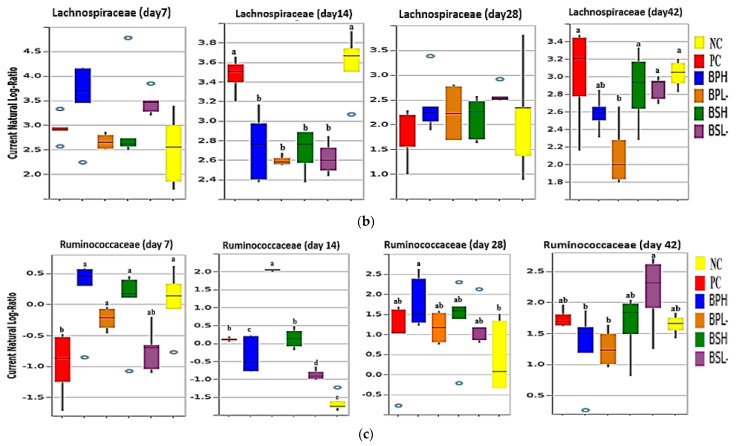
Plots showing relative frequency of the microbial taxonomic composition of different phyla (**a**) and the microbial relative abundance of the family Lachnospiraceae (**b**) and the family Ruminococcaceae (**c**) in response to treatments at 7, 14, 28, and 42 days of age. The abundance of Lachnospiraceae and Ruminococcaceae was calculated as the natural log-ratios. The Ruminococcus was used as a reference frame in the log ratio test. Chickens were fed dietary treatments as described in Table 1. Boxes in panels b and c show the medians/quartiles of treatment samples, and the error bars extend to the most extreme values within the 1.5 interquartile ranges (*n* = 4 or 5). ^a–e^ Different letters mean significant differences among groups (*p* < 0.05).

To study changes of microbial taxa by probiotics, the abundance of microbiota was examined at days 7, 14, 28, and 42 of the broiler’s life due to the importance of these time points in the development of stable microbiota. From the cecal samples from all of the treatments and time points, Firmicutes, Actinobacteria, Proteobacteria, and Bacteroidetes were the dominant phyla, accounting for a major part of the total sequence reads (Figure 1a). The families of Lachnospiraceae and Ruminococcaceae from the Phylum Firmicutes accounted for around 70% of the microbiota and showed higher differences among the treatment groups at day 14 than they did at the other timepoints (Figure 1b,c).

The microbial abundance of Ruminococcaceae and Lachnospiraceae was not significantly different among different treatments at day 7, except for the PC group, which reduced the abundance of the Ruminococcaceae in compared to the NC group. The family of Lachnospiraceae showed the highest abundance in the NC and PC groups at day 14 followed by a comparative decline in abundance at days 28 and 42 (Figure 1b). In contrast, the Ruminococcaceae family had the lowest abundance at day 14 in the NC and PC groups followed by increase in abundance at days 28 and 42 (Figure 1c). Conversely, the probiotic groups had a significantly (*p* < 0.05) lower abundance of Lachnospiraceae (Figure 1b) and a higher abundance of Ruminococcaceae (Figure 1c) at day 14 compared to the NC group, which became comparable to the NC group at days 28 and 42 in all of the probiotic groups except for in the BPH group at day 28, which had a higher abundance of Ruminococcaceae than the NC group and the BPL group at day 42, which had a lower abundance of Lachnospiraceae than the NC group. The abundance of Ruminococcaceae was also significantly higher (*p* < 0.05) in the PC group at day 14 compared to the NC group, which became insignificant at day 28 and onward in comparison with the NC group (Figure 1c). These changes were further analyzed in the detected genera that covered at least 2% of the features. Differences in the composition of the microbiota among the treatment groups at the Genus level were presented in Table 3. As shown in Table 3a, at day 7, the abundance of *Oscillospira* (BPL and BSL), *Faecalibacterium* (BPH), and *Butyricicoccus* (BSH) was significantly high, while the abundance of *Blautia* (BPH and BPL) and Enterobacteriaceae (BPH and BSL) was substantially lower in the probiotic groups when compared to the NC group. The PC group showed a significant increase in the abundance of Enterobacteriaceae and a decline in *Oscillospira* in comparison with the NC group at day 7. The abundance of different genera from family Ruminococcaceae, such as *Ruminococcus* (BPL, BPH, BSL, and BSH), *Oscillospira* (BPL, BSL, and BSH), *Faecalibacterium* (PC, BPL, BPH, BSL, and BSH), *Butyricicoccus* (BSL and BSH), and *Subdoligranulum* (BPL), was significantly higher at day 14 (Table 3a) compared to the NC group but became non-significant in many groups by days 28 and 42 (Table 3b), with the exception of *Ruminococcus* (BPL), *Oscillospira* (PC, BPL, BPH, BSL, and BSH), and *Faecalibacterium* (BPH and BSH) at day 28 and *Ruminococcus* (BPL) and *Subdoligranulum* (BSH) at day 42, where the abundance of these groups remained high. The abundance of *Subdoligranulum* and *Butyricicoccus* was lower in the PC group than in the NC group at day 14 and was similar to the NC group at days 28 and 42. In contrast, the abundance of genera from the family Lachnospiraceae, such as *Ruminococcus* (PC, BPL, BPH, BSL, and BSH), *Blautia* (PC, BPL, and BPH), *Coprococcus* (PC, BPL, BPH, BSL, and BSH), and *Dorea* (PC), was significantly lower than it was in the NC group (*p* < 0.05) at day 14 but became comparable to the NC group at days 28 and 42 in all of the groups, except *Blautia* (for BPH and BSH) at day 28 and *Coprococcus* (PC, BPH and BSL) at days 28 and 42, where the abundance of these groups remained lower than it was in the NC group. The abundance of *Dorea* (PC and BSL) and *Blautia* (BPL) was higher than it was in the NC group at day 42. The abundance of *Dorea* in the BPL and BPH groups was significantly higher (*p* < 0.05) at day 14 and became insignificant at days 28 and 42. The abundance of other genera such as *Lactobacillus*, a member of the Lactobacillaceae family, and *Bifidobacterium*, a member of the family Bifidobacteriaceae, was significantly greater (*p* < 0.05) in the BPH, BSH, and BPL groups at day 14 but was not different from the NC group at days 28 and 42. The abundance of *Enterococcus* (BPL and BSL), *Sutterella* (BSH and BSL), and Erysipelotrichaceae (PC and BPL) was lower at day 14 in different groups and became equivalent to the NC group at days 28 and 42, except in case of *Sutterella* (PC, BPH, BSH and BSL) at days 28 and 42 and *Enterococcus* (BPL) at day 42. The abundance of *Enterococcus* was high in the PC group at day 14, which was detected to be equivalent to the NC group at days 28 and 42. Interestingly, the abundance of the Enterobacteriaceae family was significantly lower in the probiotic groups but was significantly higher in the PC group at days 7, 14, and 28. At day 42, all of the groups became insignificant in comparison with the NC group.

## 4. Discussion

Our results revealed that probiotics alleviated an age-related (compared to the NC group) and antibiotic induced (compared to the PC group) drop in the alpha diversity mainly through the improvement in richness in younger birds before day 14. Our results are in agreement with earlier studies that used probiotics to alleviate dysbiosis caused by antibiotics. The study of Engelbrektson et al. [27] reported a lessening in antibiotic-induced dysbiosis using a probiotic preparation carrying bacterial populations of *bifidobacteria* and *lactobacilli* in humans. In chickens, Pereira et al. [13] reported that *B. subtilis*-based probiotics prevented an antibiotic-induced reduction in microbial richness and diversity. In another study, Oh et al. [28] reported improvements in the functional parameters of microbiota following probiotic supplementation with antibiotic therapy. Our study results also displayed that improving bacterial diversity in younger birds (before d14) was dependent on the strain and the dose of the probiotic. The *B. subtilis* probiotics (BSL and BSH) maintained the diversity between days 7 and day 14, while the BPL group exhibited a reduction in the alpha diversity of the microbiota at day 14 compared to the BPH group, where it increased significantly at day 14. Nevertheless, BPL induced a reduction in the alpha-diversity on day 14, which was quickly recovered at day 28. A similar drop in the microbial diversity in response to probiotics in broiler chickens was also observed by Trela et al. [29], who reported a decrease in the biodiversity indices, Shannon and Simpson, of crop and jejunum microbiota in response to the *B. licheniformis* probiotic. These observations highlight that probiotics can help in the prevention of a decline in the microbial diversity in cecal microbiota, in strain- and dose-dependent manner. In addition, beta diversity analyses support the notion that changes in the cecal microbiota due to probiotic groups were visible in the early weeks of life and were most prominent at day 14, and these changes became insignificant after day 28.

The maturation of microbiota can be reflected in alpha diversity, beta diversity, changes in composition of microbiota, and functional genes. Our results also demonstrated that probiotics help cecal microbiota achieve early maturation at day 14 through an increase in the abundance of the core members of the family Ruminococcaceae such as *Ruminnococcus*, *Oscillospira*, *Faecalibacterium,* and *Butyricicoccus*. Growth in the family Ruminococcaceae happens at the cost of members of the family Lachnospiraceae such as *Ruminococcus*, *Blautia,* and *Coprococcus*. The bacterial fermentation of indigestible polysaccharides into short chain fatty acids (SCFA) is one of main functions in the caecum. SCFA are utilized by intestinal epithelial cells. The bacterial populations that are active in fermentation belong to certain families of Firmicutes such as Lachnospiraceae or Ruminococcaceae [18]. After day 21, the intestinal microbota become stable and mature as variations in its structure and composition become lessened [18,30]. The members of the family Lachnospiraceae, *Blautia* and *Ruminococcus*, are reported as the dominant bacterial population in the dynamic microbiota at the early days of life [17,19], while members of the family Ruminococcaceae, *Faecalibacterium*, dominate in mature microbiota at day 21 and onward [18,19]. Our results reflected that *Bacillus subtilis* and *Bacillus pumilus* improved the strictly anaerobic population of the family Ruminococcaceae in early weeks of life, which is considered a major part of mature microbiota and have beneficial effects on the host physiology.

The early maturation of microbiota is beneficial to host’s immune functions, as we reported previously [12], in which the improvement in intestinal integrity and in the function and activation of anti-inflammatory T regulatory cells were observed in response to *B. subtilis* and *B. pumilus* probiotics at day 14 of broiler life. Our results are also supported by results from others. The *Faecalibacterium prausnitzii* is thought to have an anti-inflammatory effect [31] and improved intestinal barrier function in a mouse IBD model [32]. Massacci et al. [33] reported an increased abundance of *Faecalibacterium prausnitzii* in response to *Saccharomyces cerevisiae boulardii*, which also potentially improved gut health and reduced *Campylobacter jejuni* excretion in broiler birds.

The bacterial population related to the family of Enterobacteriaceae was significantly higher in the PC group but was substantially lower in the probiotic groups in the current study. This bacterial family is important in poultry production, as it contains many pathogens with antimicrobial resistance such as extended-spectrum beta-lactamase genes [34]. Byndloss [35] reported that antibiotics decreased the butyrate producing obligate anaerobic bacterial population, such as Clostridia (Ruminococcaceae and Lachnospiraceae), which are responsible for the maintenance of physiologic hypoxia on intestinal epithelial surfaces. A reduction in hypoxic conditions ease oxygen tolerant facultative anaerobes, such as Enterobacteriaceae, to grow fast and to overgrow other microbial populations. Thus, the probiotics (BPL, BPH and BSH) in this study may improve hypoxic conditions at the epithelial level by supporting the growth of short chain fatty acid producing (butyrate) bacteria such as members of the family Ruminococcaceae, which prevent the colonization of the Enterobacteriaceae population containing disease-causing bacteria. These results highlight that *Bacillus* probiotics favor the health promoting microbial population and play a protective role for the host.

The bacterial populations of *Bifidobacterium* (BPL) and *Lactobacillus* (BPH and BSH) were higher in specific probiotic groups at day 14. *Bifidobacteria* provides a substrate for bacteria that constitute the mature configuration of stable microbiota. Earlier studies reported that certain strains of Bifidobacteria secrete exopolysaccharides, a complex carbohydrate that acts as a substrate for mature microbiota such as *Faecalibacterium prausnitzii* [36] and *Bacteroides fragilis* [37]. In addition to promoting the growth of mature cecal microbiota, Bifidobacteria are reported to have a role in the intestinal barrier functions [38] and in the maturation and balancing of immune cells [39]. This suggests that an early rise in the population of strains of *Bifidobacterium* could help in the development and the activation of mature microbiota in hosts. *Lactobacillus* bacteria were higher in the BPH and BSH groups. *Lactobacillus* species are considered to be beneficial to the host in terms of their potential roles in decreasing intestinal pathogens through competitive exclusion, the production of bacteriocins, and antagonistic activities [40].

In this study the Gram-positive bacterial populations such as *Lactobacillus* may be underrepresented due to the presence of monensin in the feed as the anticoccidial in all treatment groups. By nature, monensin belongs to monovalent carboxylic ionophore group of anticoccidials and is naturally produced by the fermentation of Streptomyces species. It interacts with the sporozoite stage of coccidial parasites in the intestinal lumen and interferes with ion transportation across the coccidial membrane, which leads to the death of the parasite [41]. The effect of monensin on intestinal microbiota is not very clear. It is generally considered that Gram-negative bacteria are intrinsically resistant to monensin, while Gram-positive bacteria show susceptibility to monensin [42]. In an in vitro study, the *Lactobacillus* population decreased in response to monensin [43]. However, in in vivo studies, either no significant effect of monensin on intestinal microbiota was seen when comparing the monensin control with the negative control [44] or some Gram-positive microbial genera decreased in abundance, while others were significantly enriched [45]. Considering that monensin was applied across all of the treatment groups in this study, the effects of the probiotics could be minimally compromised. However, the effect of monensin on the action of probiotics on poultry intestinal microorganisms requires further investigation.

## 5. Conclusions

In summary, the probiotic groups efficiently promoted the earlier maturation of cecal microbiota. The effects were strain and dose specific. *B. pumilus* and *B. subtilis* improved health promoting microorganisms such as members of Ruminococcaceae, *Lactobacillus,* and *Bifidobacterium* while virginiamycin increased the abundance of Enterobacteriaceae, which is linked with entero-pathogens. These results will set the stage for the design of microbiota-based interventions to promote production and health or to prevent diseases in chickens.

## Figures and Tables

**Table 1:** Effects of dietary treatments on microbial richness (**a**), evenness (**b**), and diversity (**c**) in broiler chickens at days 7, 14, 28, and 42 of age.

a. Effects of dietary treatments on microbial richness (observed operational taxonomic units).								
**Groups/Time**	**Mean Day-7**	**SEM**	**Mean Day-14**	**SEM**	**Mean Day-28**	**SEM**	**Mean Day-42**	**SEM**
NC	110 ^3^	11.9	100 ^b,3^	13.3	188 ^2^	11.9	241 ^1^	13.3
PC	92 ^2^	11.9	109 ^b,2^	13.3	188 ^1^	11.9	202 ^1^	13.3
BPL	116 ^3^	13.3	172 ^a,2^	13.3	231 ^1^	13.3	228 ^1^	13.3
BPH	118 ^2^	11.9	163 ^a,1,2^	11.9	202 ^1^	11.9	198 ^1^	13.3
BSL	135 ^2^	11.9	172 ^a,2^	13.3	234 ^1^	11.9	249 ^1^	11.9
BSH	106 ^2^	11.9	177 ^a,1^	13.3	221 ^1^	11.9	199 ^1^	13.3
b. Effects of dietary treatments on microbial evenness (Pielou index) at days 7, 14, 28, and 42.								
**Groups/Time**	**Mean Day-7**	**SEM**	**Mean Day-14**	**SEM**	**Mean Day-28**	**SEM**	**Mean Day-42**	**SEM**
NC	0.77 ^ab,1^	0.025	0.66 ^ab,2^	0.029	0.75 ^ab,1,2^	0.025	0.69 ^1,2^	0.029
PC	0.65 ^c,2^	0.025	0.63 ^bc,2^	0.029	0.77 ^a,1^	0.025	0.61 ^2^	0.029
BPL	0.77 ^ab,1^	0.029	0.52 ^c,2^	0.029	0.74 ^ab,1^	0.029	0.73 ^1^	0.029
BPH	0.68 ^bc,1,2^	0.025	0.77 ^a,1^	0.025	0.68 ^ab,2^	0.025	0.64 ^2^	0.029
BSL	0.76 ^ab,1^	0.025	0.68 ^ab,1,2^	0.029	0.71 ^ab,12^	0.025	0.63 ^2^	0.025
BSH	0.80 ^a,1^	0.025	0.72 ^ab,1,2^	0.029	0.66 ^b,2,3^	0.025	0.61 ^3^	0.029
c. Effects of dietary treatments on microbial diversity (Shannon index) at days 7, 14, 28, and 42.								
**Groups/Time**	**Mean Day-7**	**SEM**	**Mean Day-14**	**SEM**	**Mean Day-28**	**SEM**	**Mean Day-42**	**SEM**
NC	5.30 ^a,1^	0.218	4.39 ^bcd,2^	0.244	5.67 ^1^	0.218	5.46 ^ab,1^	0.244
PC	4.24 ^b,2^	0.218	4.25 ^cd,2^	0.244	5.77 ^1^	0.218	4.69 ^ab,2^	0.244
BPL	5.25 ^a,1^	0.244	3.88 ^d,2^	0.244	5.81 ^1^	0.244	5.69 ^a,1^	0.244
BPH	4.67 ^ab,2^	0.218	5.66 ^a,1^	0.218	5.17 ^1,2^	0.218	4.89 ^ab,1,2^	0.244
BSL	5.37 ^a^	0.218	5.04 ^abc^	0.244	5.59	0.218	5.02 ^ab^	0.218
BSH	5.39 ^a^	0.218	5.36 ^ab^	0.244	5.11	0.218	4.65 ^b^	0.244

Chickens were fed a basal diet (NC), a basal diet with antibiotic as a positive control (PC), a basal diet with a low-dose of *B. pumilus* (BPL), a basal diet with a high-dose of *B. pumilus* (BPH), a basal diet with a low-dose of *B. subtilis* (BSL), and a basal diet with a high-dose of *B. subtilis* (BSH). ^a–d^ Different letters in superscript mean significant differences between groups in columns, while ^1–3^ different numbers in superscript mean significant differences within groups at different time points (days 7, 14, 28, and 42) in rows (*p* < 0.05) (*n* = 4 or 5).

**Table 2:** Pairwise microbial community dissimilarity (beta diversity) in response to dietary treatments between groups at days 7 (**a**), 14 (**b**), 28 (**c**), and 42 (**d**) and within groups between different timepoints (**e**).

a. Pairwise microbial dissimilarity between groups at day 7.						
**Treatment Groups**	**NC**	**PC**	**BPL**	**BPH**	**BSL**	**BSH**
NC	1	0.04	0.04	0.03	0.04	0.04
PC		1	0.04	0.03	0.04	0.04
BPL			1	0.03	0.04	0.04
BPH				1	0.03	0.06
BSL					1	0.04
BSH						1
b. Pairwise microbial dissimilarity between groups at day 14.						
**Treatment Groups**	**NC**	**PC**	**BPL**	**BPH**	**BSL**	**BSH**
NC	1	0.07	0.07	0.04	0.07	0.03
PC		1	0.06	0.03	0.08	0.04
BPL			1	0.03	0.06	0.03
BPH				1	0.03	0.03
BSL					1	0.03
BSH						1
c. Pairwise microbial dissimilarity between groups at day 28						
**Treatment Groups**	**NC**	**PC**	**BPL**	**BPH**	**BSL**	**BSH**
NC	1	0.24	0.03	0.06	0.03	0.03
PC		1	0.18	0.14	0.11	0.14
BPL			1	0.24	0.04	0.04
BPH				1	0.24	0.79
BSL					1	0.09
BSH						1
d. Pairwise microbial dissimilarity between groups at day 42.						
**Treatment Groups**	**NC**	**PC**	**BPL**	**BPH**	**BSL**	**BSH**
NC	1	0.04	0.29	0.13	0.13	0.14
PC		1	0.04	0.22	0.17	0.64
BPL			1	0.56	0.13	0.41
BPH				1	0.28	0.56
BSL					1	0.78
BSH						1
e. Pairwise microbial dissimilarity within groups between days 7 and 14, 14 and 28, and 28 and 42.						
**Groups/Time Points**	**Day 7 vs. Day 14**		**Day 14 vs. Day 28**		**Day 28 vs. Day 42**	
NC	0.04		0.03		0.03	
PC	0.03		0.14		0.03	
BPL	0.05		0.04		0.27	
BPH	0.04		0.03		0.52	
BSL	0.03		0.03		0.03	
BSH	0.03		0.05		0.53	

The data of weighted UniFrac distance matrix were analyzed through a PERMANOVA test, and the results were corrected for significance through the Benjamini–Hochberg FDR method (q-values). q-values equal to or less than 0.05 were considered statistically significant (*n* = 4 or 5). Chickens were fed dietary treatments as described in Table 1.

**Table 3:** Effects of dietary treatments on the relative abundance of cecal microbiota at days 7, 14, 28 and 42.

a. Effects of dietary treatments on the relative abundance of cecal microbiota at days 7 and 14.													
		**Treatments (Day-7) ^1^**						**Treatments (Day-14) ^1^**					
**Family**	**Genus**	**PC**	**BPH**	**BPL**	**BSH**	**BSL**	**NC**	**PC**	**BPH**	**BPL**	**BSH**	**BSL**	**NC**
Ruminococcaceae	*Ruminococcus*	−2.9	−3.5	−2.7	−3.0	−3.5	−2.5	−3.5 ^b^	**−2.7 ^a^**	**−2.6 ^a^**	**−2.7 ^a^**	**−2.6 ^a^**	−3.6 ^b^
	*Oscillospira*	−4.3 ^d^	−3.8 ^c^	**−2.9 ^a^**	−3.4 ^b^	**−2.8 ^a^**	−3.5 ^bc^	−3.9 ^bc^	−3.5 ^abc^	**−2.9 ^a^**	**−2.8 ^a^**	**−3.2 ^ab^**	−4.1 ^c^
	*Faecalibacterium*	−6.4 ^b^	**−1.2** **^a^**	−6.0 ^b^	−6.9 ^b^	−6.5 ^b^	−6.3 ^b^	**−0.7 ^a^**	**−3.7 ^cd^**	**−2.9 ^b^**	**−3.6 ^c^**	**−4.0 ^d^**	−6.2 ^e^
	*Butyricicoccus*	−4.2 ^b^	−4.8 ^b^	*	**−2.2 ^a^**	−4.6 ^b^	−4.4 ^b^	**−6.5 ^c^**	−4.2 ^ab^	−4.4 ^ab^	**−3.7 ^a^**	**−4.0 ^a^**	−5.1 ^b^
	*Subdoligranulum*	*	*	*	*	*	*	**−7.0 ^c^**	−3.3 ^b^	**0.28 ^a^**	−2.7 ^b^	−4.2 ^b^	−3.4 ^b^
Lachnospiraceae	*Ruminococcus*	0.15	−0.21	0.24	−0.01	0.29	−0.05	**−0.1 ^d^**	**0.3 ^c^**	**−2.0 ^e^**	**−0.1 ^d^**	**0.9 ^b^**	1.7 ^a^
	*Blautia*	−0.9 ^b^	**−1.6 ^a^**	**−1.4 ^a^**	−0.4 ^bc^	−0.3 ^c^	−0.7 ^bc^	**−2.7 ^c^**	**−1.2 ^b^**	**−2.7 ^c^**	−0.7 ^ab^	0.1 ^a^	−0.1 ^a^
	*Coprococcus*	−0.6 ^b^	−2.5 ^b^	−1.3 ^b^	−0.9 ^b^	−0.7 ^b^	−1.6 ^ab^	**−2.6 ^c^**	**−1.0 ^b^**	**−3.7 ^d^**	**−1.5 ^b^**	**−1.1 ^b^**	−0.4 ^a^
	*Dorea*	−3.4	−4.3	−4.1	−3.4	−3.6	−4.3	**−5.2 ^d^**	**−3.4 ^b^**	**−2.6 ^a^**	−3.8 ^bc^	−4.4 ^c^	−4.1 ^c^
	*Clostridium*	−5.4	−6.1	−5.4	−5.9	−5.7	−5.4	−5.3 ^a^	−6.1 ^ab^	**−7.3 ^b^**	−4.8 ^a^	−6.5 ^ab^	−5.2 ^a^
Lactobacillaceae	*Lactobacillus*	−1.01	−1.81	−0.04	−1.18	−0.74	−1.08	*	**−2.7 ^b^**	−4.8 ^c^	**−0.9 ^a^**	−5.1 ^c^	−5.2 ^c^
^2^ Erysipelotrichaceae	-	−2.6	−3.2	−2.3	−1.9	−2.0	−2.6	**−4.0 ^b^**	−2.5 ^a^	**−4.4 ^b^**	−2.7 ^a^	−2.6 ^a^	−2.8 ^a^
Enterococcaceae	*Enterococcus*	−0.4 ^ab^	−1.8 ^abc^	0.2 ^a^	−1.8 ^bc^	−3.0 ^c^	−1.2 ^a–c^	**−3.3 ^a^**	−4.5 ^b^	**−5.2 ^c^**	−4.4 ^b^	**−6.0 ^d^**	−4.0 ^b^
Bifidobacteriaceae	*Bifidobacterium*	*	*	*	*	*	*	−6.7 ^b^	−6.6 ^b^	**−0.6 ^a^**	−3.9 ^b^	−5.6 ^b^	−4.6 ^b^
Alcaligenaceae	*Sutterella*	*	*	*	*	*	*	−2.2 ^ab^	−4.0 ^ab^	−4.0 ^ab^	**−5.0 ^bc^**	**−7.2 ^c^**	−1.0 ^a^
^2^ Enterobacteriaceae	-	**−0.7** **^a^**	**−3.7** **^c^**	−2.8 ^bc^	−2.9 ^bc^	**−3.7** **^c^**	−1.9 ^b^	**−1.6 ^a^**	**−5.3 ^c^**	**−5.0 ^c^**	**−5.6 ^c^**	−2.9 ^b^	−2.5 ^b^
b. Effects of dietary treatments on relative abundance of cecal microbiota at days 28 and 42.													
		**Treatments (Day-28) ^1^**						**Treatments (Day-42) ^1^**					
**Family**	**Genus**	**PC**	**BPH**	**BPL**	**BSH**	**BSL**	**NC**	**PC**	**BPH**	**BPL**	**BSH**	**BSL**	**NC**
Ruminococcaceae	*Ruminococcus*	−1.8 ^ab^	−1.8 ^ab^	**−1.1** **^a^**	−2.1 ^b^	−2.6 ^b^	−2.3 ^b^	−3.0 ^b^	−2.6 ^ab^	**−2.1 ^a^**	−2.9 ^b^	−2.8 ^b^	−3.0 ^b^
	*Oscillospira*	**−2.7** **^a^**	**−2.6** **^a^**	**−2.4** **^a^**	**−2.5** **^a^**	**−2.8** **^a^**	−3.4 ^b^	−2.6	−2.9	−2.6	−3.2	−2.6	−2.8
	*Faecalibacterium*	−1.66 ^b^	**0.03** **^a^**	−0.68 ^ab^	**−0.07** **^a^**	−0.46 ^ab^	−1.7 ^b^	0.6 ^a^	0.2 ^ab^	−0.4 ^b^	0.3 ^ab^	0.5 ^ab^	0.01 ^ab^
	*Butyricicoccus*	−4.0	−3.8	−4.5	−4.5	−3.6	−4.5	−4.9 ^ab^	−4.3 ^ab^	−3.6 ^a^	−5.1 ^b^	−4.6 ^ab^	−3.9 ^ab^
	*Subdoligranulum*	−4.1	−4.8	−4.5	−4.6	−6.3	−4.9	−4.1 ^ab^	−5.7 ^b^	−5.1 ^b^	**−2.8 ^a^**	−5.3 ^b^	−5.2 ^b^
Lachnospiraceae	*Ruminococcus*	−0.9	−1.7	−1.2	−1.3	−1.2	−0.5	−1.7 ^ab^	−1.3 ^a^	−1.3 ^a^	−1.6 ^ab^	−2.1 ^b^	−1.6 ^ab^
	*Blautia*	−1.5 ^ab^	**−2.9 ^ c^**	−1.2 ^ab^	**−2.5** **^bc^**	−1.4 ^ab^	−1.0 ^a^	−2.9 ^ab^	−2.4 ^ab^	**−2.0 ^a^**	−2.5 ^ab^	−2.6 ^ab^	−3.2 ^b^
	*Coprococcus*	**−2.6** **^bc^**	**−3.3** **^c^**	−2.1 ^ab^	−2.2 ^ab^	**−2.5** **^b^**	−1.7 ^a^	**−3.5 ^b^**	**−3.4 ^b^**	−2.4 ^ab^	−3.0 ^ab^	**−3.5 ^b^**	−2.3 ^a^
	*Dorea*	−3.8	−3.6	−3.6	−3.7	−3.3	−3.6	**−3.1 ^a^**	−4.7 ^b^	−3.8 ^ab^	−4.4 ^b^	**−3.1 ^a^**	−4.3 ^b^
	*Clostridium*	−3.9	−4.5	−4.2	−4.5	−4.2	−4.1	−4.3	−3.8	−3.6	−4.2	−3.5	−3.5
Lactobacillaceae	*Lactobacillus*	−4.5	−3.5	−4.1	−4.6	−3.9	−3.4	−4.4 ^b^	−2.2 ^a^	−2.3 ^a^	−4.0 ^ab^	−4.5 ^b^	−3.6 ^ab^
^2^ Erysipelotrichaceae	-	−3.1	−3.9	−3.8	−3.6	−3.2	−3.5	−4.6	−3.1	−3.2	−4.1	−3.8	−3.9
Enterococcaceae	*Enterococcus*	−4.1	−5.6	−5.3	−5.6	−5.2	−3.9	−5.5 ^ab^	−5.7 ^ab^	**−4.5 ^a^**	−6.8 ^b^	−6.1 ^ab^	−6.4 ^b^
Bifidobacteriaceae	*Bifidobacterium*	−2.4	−6.0	−5.4	−5.0	−4.1	−3.1	−3.0	−5.1	−3.0	−2.4	−2.1	−1.9
Alcaligenaceae	*Sutterella*	**−3.7** **^c^**	**−3.7** **^c^**	−1.9 ^ab^	**−3.4** **^c^**	**−2.8** **^bc^**	−1.4 ^a^	**−4.5 ^c^**	**−3.4 ^bc^**	−2.4 ^ab^	**−3.8 ^c^**	**−3.8 ^c^**	−1.7 ^a^
^2^ Enterobacteriaceae	-	**−1.2** **^a^**	−3.0 ^b^	−4.2 ^cd^	**−5.1** **^d^**	−4.3 ^cd^	−3.8 ^bc^	−4.5	−5.6	−3.8	−4.3	−4.7	−5.1

The relative abundance of microbiota was calculated as the natural log-ratios at days 7 and 14 (a) and days 28 and 42 (b). The Ruminococcaceae was used as a reference frame for the features of all of the other microbial families, while Lachnospiraceae was used as a reference frame for the features of the family Ruminococcaceae. ^1^ Chickens were fed dietary treatments as described in Table 1. Numbers in green, red, and black represents high, low, and no significant difference in abundance from the NC group, respectively. ^2^ Genera not detected. * Values not detected. ^a–d^ Different letters mean significant differences among groups (*p* < 0.05). Significance level was adjusted for multiple comparison through Duncan test (*n* = 4 or 5).

## Data Availability

The data presented in this study can be provided on request from the corresponding author.
